# Association between total serum cholesterol and depression, aggression, and suicidal ideations in war veterans with posttraumatic stress disorder: a cross-sectional study

**DOI:** 10.3325/cmj.2014.55.520

**Published:** 2014-10

**Authors:** Maja Vilibić, Vlado Jukić, Mirna Pandžić-Sakoman, Petar Bilić, Milan Milošević

**Affiliations:** 1 Department of Biological Psychiatry and Psychogeriatrics, Vrapče University Psychiatric Hospital, Zagreb, Croatia; 2 Department of Forensic Psychiatry, Vrapče University Psychiatric Hospital, Zagreb, Croatia; 3 Department of Social Psychiatry, Vrapče University Psychiatric Hospital, Zagreb, Croatia; 4 Department for Diagnostic and Intensive Treatment, Vrapče University Psychiatric Hospital, Zagreb, Croatia; 5 Andrija Štampar School of Public Health, Zagreb, Croatia

## Abstract

**Aim:**

To investigate the relationship between total serum cholesterol and levels of depression, aggression, and suicidal ideations in war veterans with posttraumatic stress disorder (PTSD) without psychiatric comorbidity.

**Methods:**

A total of 203 male PTSD outpatients were assessed for the presence of depression, aggression, and suicidality using the 17-item Hamilton Depression Rating Scale (HAM-D_17_), Corrigan Agitated Behavior Scale (CABS), and Scale for Suicide Ideation (SSI), respectively, followed by plasma lipid parameters determination (total cholesterol, high density lipoprotein [HDL]-cholesterol, low density lipoprotein [LDL]-cholesterol, and triglycerides). PTSD severity was assessed using the Clinician-Administered PTSD Scale for DSM-IV, Current and Lifetime Diagnostic Version (CAPS-DX) and the Clinical Global Impressions of Severity Scale (CGI-S), before which Mini-International Neuropsychiatric Interview (MINI) was administered to exclude psychiatric comorbidity and premorbidity.

**Results:**

After adjustments for PTSD severity, age, body mass index, marital status, educational level, employment status, use of particular antidepressants, and other lipid parameters (LDL- and HDL- cholesterol and triglycerides), higher total cholesterol was significantly associated with lower odds for having higher suicidal ideation (SSI≥20) (odds ratio [OR] 0.09; 95% confidence interval [CI] 0.03-0.23], clinically significant aggression (CABS≥22) (OR 0.28; 95% CI 0.14-0.59), and at least moderate depressive symptoms (HAM-D_17_≥17) (OR 0.20; 95% CI 0.08-0.48). Association of total cholesterol and HAM-D_17_ scores was significantly moderated by the severity of PTSD symptoms (*P* < 0.001).

**Conclusion:**

Our results indicate that higher total serum cholesterol is associated with lower scores on HAM-D_17_, CABS, and SSI in patients with chronic PTSD.

Posttraumatic stress disorder (PTSD) is one of the few mental disorders with a clearly identifiable cause. It is an anxiety disorder caused by exposure to a traumatic event that presented a threat to the physical integrity of persons themselves or other people in their surroundings (1). Key neurochemical PTSD features include altered catecholamines regulation, alterations in serotonergic system, and alterations in systems of aminoacids, peptides, and opioid neurotransmitters (2).

Associations between serum lipids and various psychiatric disorders and some behavioral aspects (like aggressive behavior) and/or suicidality have been widely explored. Lower total cholesterol levels were predominantly found in patients with major depressive disorder (MDD) (3-9). Significantly higher high-density lipoprotein cholesterol (HDL-cholesterol) levels were found in depressive patients than in controls (7). Some studies found significantly lower HDL-cholesterol levels (10) and a lower HDL-cholesterol/total cholesterol ratio (5) in patients with MDD than in controls.

A negative correlation (11-13) between serum cholesterol level and aggressive behavior was also found, confirming the cholesterol-serotonergic hypothesis of aggression (14,15). Inadequate cholesterol intake could lead to decreased central serotonin activity, which is associated with an increased risk for impulsive-aggressive behavior (14-18). Depression (19-21) and aggression are well-known suicidality risk factors (15,22).

The correlation between hypocholesterolemia, decreased central serotonin activity, increased depressive potential, and increased suicidality risk (23-27) was confirmed, implicating that hypocholesterolemia might be indirectly, ie, through decreased central serotonin activity and increased depression potential (20,25,28), associated with an increased suicidality risk (15,19-24,26,27). In patients with anxiety disorders other than PTSD, like panic disorder (PD), lower HDL-cholesterol and higher very low density lipoprotein cholesterol (VLDL-cholesterol) levels were found to be associated with higher suicide ideations/risk (29). Significantly lower serum total cholesterol and LDL cholesterol levels were found in suicidal patients with PD than in control subjects (30).

Hypercholesterolemia was found to be associated with chronic, war-related PTSD (31-34). In a study from Bosnia and Herzegovina, not only hypercholesterolemia but also increased VLDL- and HDL-cholesterol levels were found in war veterans with PTSD in comparison with war veterans without psychiatric disorders (35). A Croatian study found no significant differences in the total serum cholesterol level, LDL-, and HDL-cholesterol between war veterans with PTSD, war veterans without PTSD, and healthy volunteers (36).The aim of this study was to investigate the relationship between serum cholesterol and levels of depression, aggression, and suicidal ideations in war veterans with PTSD free of other psychiatric premorbidity and comorbidity.

## Patients and methods

### Patients

This single-center, cross-sectional study included patients consecutively sampled from the pool of veterans involved in outpatient PTSD program at the Department for Biological Psychiatry and Psychogeriatrics and the Department for Diagnostic and Intensive Treatment, University Psychiatric Hospital Vrapče, Zagreb, Croatia. The study was approved by the Ethics Committee of the Vrapče University Psychiatric Hospital. The participants were not reimbursed for their participation in the study and received no benefit in their treatment in comparison with outpatients with PTSD treated in the same hospital but not included in the study. Participants were consecutively recruited from January 2007 till November 2012. Out of 427 examined potential study participants, 10 refused to participate and 214 were not eligible for the study. Out of 203 included study participants, all 203 finished the study.

The study included adult men with chronic, war-related PTSD who met all of the following inclusion criteria: (i) signed an informed consent prior to any study procedure, (ii) male sex, (iii) age between 18 and 65 years, (iv) outpatient status, and (v) confirmed PTSD according to both ICD-10 (37) and DSM-IV (1) criteria. The exclusion criteria were (i) premorbid or comorbid psychiatric disorders (including alcohol and other dependence disorders, eating disorders, or any other psychiatric disorder or condition different from PTSD), (ii) significant somatic comorbidity (particularly those diseases and disorders, like metabolic, with significant influence on cholesterol and other serum lipids metabolism including malnutrition, malabsorption, chronic infections, consumptive disorders, hyper- and hypothyreosis, diabetes mellitus), (iii) BMI lover than 20 kg/m^2^, and (iv) taking medicaments with significant influence on serum lipids metabolism (particularly hypolipemics).

### Psychiatric evaluation

All patients met the ICD-10 PTSD criteria, which is the official classification used in Croatian psychiatric practice. PTSD severity was assessed by DSM-IV-based Clinician-Administered PTSD Scale, Current and Lifetime Diagnostic Version (CAPS-DX) (38) and the Clinical Global Impressions of Severity (CGI-S) scale (39), before which Mini-International Neuropsychiatric Interview (MINI) (40) was administered. MINI, a structured diagnostic interview, was used to confirm that each patient met DSM-IV PTSD criteria with the exclusion of psychiatric comorbidity and premorbidity.

After three consecutively administered scales (MINI, CAPS-DX, and CGI-S), the following standardized psychometric instruments/questionnaires were used: Corrigan Agitated Behavior Scale (CABS) (41), 17-item Hamilton Depression Rating Scale (HAM-D_17_) (42), and the Scale for Suicide Ideation (SSI) (43). CABS was used for assessment of the current aggression level (aggressive behaviors), HAM-D_17_ for depression potential (subsyndromal depressive states), and SSI for suicidality potential (suicidal ideations). All six scales were applied by experienced psychiatrists, licensed for their administration. The scales were used in their original formats and language, whereas the interview was conducted in Croatian. HAM-D_17_ is one of the most frequently used clinician-administered depression assessment scales. It has 17 items; the score of 0-6 points is considered normal, while the score of 7-16 points indicates mild, 17-24 points moderate, and ≥25 points severe depression. Scores ≥17 points usually indicate clinically significant levels of depressive symptoms (42). Inter-rater reliability for HAM-D_17_ is high, ranging from 0.80 to 0.98 for the total score rating. The test-retest reliability is also high, about 0.81. The validity of the HAM-D_17_ ranges from 0.65 to 0.90, with global measures of depression severity (44). In this study,HAM-D_17_ reliability calculated as internal consistency (Cronbach’s α) was 0.66. The Guttman Split-Half Coefficient of reliability was 0.72 and the Spearman-Brown coefficient for unequal length scale half was 0.72.

CABS is an observational, clinician administered, as opposed to self-report, scale. Original validation study showed that trained therapists can use the scale with sufficient reliability and validity when based on therapists’ 30-minute observation periods (41). Each of the 14 items is rated on a 4-point rating scale and the total score is calculated by adding up the rating on each of 14 items. Scores of ≥22 usually indicate a clinically significant result (41). In this study, Cronbach’s coefficient of internal CABS scale consistency was 0.96. The Guttman Split-Half Coefficient of reliability was 0.97 and the Spearman-Brown coefficient for unequal length scale half was 0.97.

The SSI is a 19-item clinician administered scale, widely used to quantify and assess suicidal intention. Each item consists of three alternative statements graded in intensity from 0 to 2. The possible range of scores is 0-38. Inter-rater reliability for SSI is high with correlations ranging from 0.83 (43) to 0.98 (45).The SSI is one of the few suicide assessment instruments to have documented the predictive validity for completed suicide. Psychiatric patients who entered the higher risk category were about seven times more likely to actually commit suicide than those who entered the lower risk category (46). In this study, Cronbach’s coefficient of internal SSI scale consistency was 0.94. The Guttman Split-Half Coefficient of reliability was 0.88 and the Spearman-Brown coefficient for unequal length scale half was 0.94. All scale administrators in this study were experienced and licensed in applying the English version of the scales, all in accordance with psychiatric scientific practice in Croatia.

### Medical assessment

General socio-demographic data: age, sex, marital and employment status, and educational level were collected by means of a clinical interview. Data on pharmacotherapy influencing serum lipids metabolism and psychopharmaceuticals taking, were collected from patients’ medical records. Data on significant somatic comorbidities were collected through physical examination and somatic anamnesis. Body weight (in kilograms, kg) and body height (in meters, m) were measured and BMI was calculated according to the following formula: BMI = weight in kg/ height in squared meters.

### Biochemical analysis

Biochemical analyses were performed at the Laboratory of the Vrapče University Psychiatric Hospital. Lipid levels were determined using commercial tests: cholesterol (enzymatic colorimetric method), HDL-cholesterol (homogeneous enzymatic colorimetric assay), LDL-cholesterol (homogeneous enzymatic colorimetric assay), and triglycerides (enzymatic colorimetric method with glycerol phosphate oxidase and 4-aminophenazone (GPO/PAP) (Roche Diagnostics, Mannheim, Germany). Concentrations were expressed in mmol/L. At two consecutive time points (separated by less than 10 but more than two days), 10 mL of blood was drawn from the cubital vein and levels of plasma lipids were determined. Blood samples were drawn between 8:00 and 9:30 am, after 12 hours of overnight fasting. They were collected using a standardized process, all in accordance with the Helsinki Declaration (47). According to the mean total cholesterol value ([value from the first sample + value from the second sample]/2), each patient was classified into one of three groups: normal group (cholesterol 3.1-5.1 mmol/L), borderline-high/high-normal group (cholesterol 5.2-6.2 mmol/L), and high cholesterol group (cholesterol >6.2 mmol/L), all in accordance with the current international guidelines (48).

### Outcomes

Three outcomes were HAM-D_17_, CABS, and SSI scales results dichotomized as to indicate at least moderate depressive symptoms (HAM-D_17_≥17), clinically significant aggression (CABS≥22), and higher – the upper quartile – suicidal ideation (SSI≥20).

### Statistical analysis

The level of statistical significance was set at *P* < 0.05 and confidence intervals at 95%. In all instances, two-tailed tests were used. Principal component analysis with extraction criterion eigenvalues >1 and Vairmax rotation with Kaiser normalization was done in order to explore the possibility of reducing dimensionality of psychometric outcomes. A univariate analysis of the relationship between lipid parameters, PTSD severity, patient's living conditions, and therapy was performed by binary logistic regression. A multivariate analysis of relationship between total cholesterol and three psychometric scales scores after adjustment for PTSD severity, age, BMI, marital status, educational level, employment status, the use of particular antidepressants, and other lipid parameters (LDL- and HDL-cholesterol and triglycerides) was done using hierarchical binary logistic regression. The moderating effect of PTSD severity on the association of total cholesterol and three psychometric scales scores was analyzed by Process (49). The CAPS value defining the region of statistically significant association of total cholesterol and three psychometric scales was assessed by Johnson-Neyman technique as implemented in the Process. Data analysis was carried out by R (50).

## Results

Out of the 427 potential study participants, 203 met all inclusion criteria and had none of the exclusion criteria (10 refused to participate and 214 were not eligible for the study). Their median (interquartile range) age was 47.0 years (43.0-54.0) and all of them were either moderately or markedly ill as measured by CGI-S (Table 1). All patients were treated with a combination of antidepressant, anxiolytic, and hypnotic treatment prescribed in standard medium dosages. By principal component analysis, 11 principal components were extracted, explaining 75% of items variance. However, the first three components (corresponding to HAM-D_17_, CABS, and SSI scales) explained 20%, 18%, and 9% of original items, respectively. All other components individually explained less than 4% of total variance. Varimax rotation with Kaiser normalization produced the structure matrix with very clear distinction between original HAMD, SSI, and CABS items. Therefore, we concluded that our three dependent variables were well discriminated, and that treating CABS, SSI, and HAM-D as separate outcomes was the most appropriate approach.

**Table 1:** Baseline characteristics of 203 male patients with posttraumatic stress disorder (PTSD)*

Characteristic	
**Age (years)^†^**	47.0 (43.0-54.0)
**PTSD symptoms severity score:**	
CAPS-DX total^†^	70.0 (65.0-76.0)
CGI-S	
moderately ill, n (%)	111 (54.7)
markedly ill, n (%)	92 (45.3)
**Living conditions:**	
Marital status	
married, n (%)	143 (70.4)
divorced, n (%)	41 (20.2)
single, n (%)	19 (9.4)
Living alone	
yes, n (%)	60 (29.6)
no, n (%)	143 (70.4)
Educational level	
elementary school, n (%)	70 (34.5)
high school, n (%)	122 (60.1)
>high school, n (%)	11 (5.4)
Employment status	
full-time, n (%)	37 (18.2)
part-time, n (%)	12 (5.9)
unemployed but able to work, n (%)	40 (19.7)
sick-leave and unemployed unable to work, n (%)	20 (9.9)
retired, n (%)	94 (46.3)
**Lipid parameters (mmol/L):^†^**	
total cholesterol	5.7 (5.0-6.0)
LDL-C	3.4 (2.9-3.8)
HDL-C	1.0 (1.0-1.4)
triglycerides	2.3 (1.5-2.9)
**BMI^†^**	26.1 (25.2-27.6)
**Therapy:**	
Antidepressants	
SSRI, n (%)	140 (69.0)
SNRI, n (%)	34 (16.7)
NaSSA, n (%)	29 (14.3)
Anxiolytics	
diazepam, n (%)	74 (36.5)
alprazolam, n (%)	58 (28.6)
clonazepam, n (%)	30 (14.8)
oxazepam, n (%)	25 (12.3)
lorazepam, n (%)	16 (7.9)
Hypnotics	
zolpidem, n (%)	159 (78.3)
nitrazepam, n (%)	44 (21.7)
**Psychometric scales:^†^**	
SSI	13.0 (7.0-19.0)
CABS	19.0 (17.0-32.0)
HAM-D_17_	18.0 (16.0-22.0)

*Abbreviations: CAPS – Clinician-Administered PTSD Scale for DSM-IV, Current and Lifetime Diagnostic Version (38); a higher score reflects higher PTSD severity; CGI-S – Clinical Global Impressions of Severity Scale (39); a higher score reflects higher PTSD severity; LDL-C – low density lipoprotein cholesterol; HDL-C – high density lipoprotein cholesterol; BMI – body mass index; SSRI – selective serotonin reuptake inhibitors; SNRI – serotonin norepinephrine reuptake inhibitors; NaSSA – noradrenergic and specific serotonergic antidepressants; SSI – Scale for Suicide Ideation (43); a higher score reflects higher suicidality potential; CABS – Corrigan Agitated Behavior Scale (41); a higher score reflects higher aggressive potential; HAM-D_17_ – Hamilton Depression Rating Scale (42); a higher score reflects higher depressive potential.

†Median (interquartile range).

Univariate binary logistic regression revealed that higher total cholesterol and LDL-cholesterol were significantly associated with lower odds for having higher suicidal ideation (SSI≥20), clinically significant aggression (CABS≥22), and at least moderate depressive symptoms (HAM-D_17_≥17) (Table 2). Higher triglycerides were associated with lower odds for higher suicidal ideation and aggression. After the adjustments for PTSD severity, age, BMI, marital status, educational level, employment status, use of particular antidepressants, and other lipid parameters (LDL and HDL cholesterol and triglycerides), higher total cholesterol was significantly associated with lower odds for having the higher suicidal ideation (SSI≥20), clinically significant aggression (CABS≥22), and at least moderate depressive symptoms (HAM-D_17_≥17) (Table 3). When the total cholesterol was removed from the equation adjusted for patients’ clinical characteristics, living conditions, and therapy, LDL-cholesterol was significantly negatively associated with lower odds for our three outcomes; HDL-cholesterol and triglycerides were significantly associated with suicidal ideation but not with aggression or depressive symptoms.

**Table 2:** Univariate association of lipid parameters with elevated SSI, CABS, HAM-D_17_ scales results (n = 203)*

	Suicidal ideation (SSI≥20)			Aggression (CABS≥22)			Depression (HAM-D_17_≥17)		
**Lipid parameters** (mmol/L):	OR	(95% CI)	*P*	OR	(95% CI)	*P*	OR	(95% CI)	*P*
total cholesterol	0.10	(0.05-0.2)	<0.001	0.33	(0.21-0.53)	<0.001	0.26	(0.16-0.42)	<0.001
LDL-C	0.27	(0.14-0.51)	<0.001	0.41	(0.24-0.68)	0.001	0.45	(0.29-0.71)	0.001
HDL-C	0.90	(0.45-1.78)	0.755	1.28	(0.72-2.29)	0.399	0.89	(0.51-1.56)	0.684
triglycerides	0.63	(0.44-0.90)	0.010	0.71	(0.53-0.95)	0.023	0.83	(0.64-1.06)	0.139

*Abbreviations: OR – odds ratio, univariate binary logistic regression; 95% CI – 95% confidence interval; LDL-C – low density lipoprotein cholesterol; HDL-C – high density lipoprotein cholesterol; SSI – Scale for Suicide Ideation (43); a higher score reflects higher suicidality potential; CABS – Corrigan Agitated Behavior Scale (41); a higher score reflects higher aggressive potential; HAM-D_17_ – Hamilton Depression Rating Scale (42); a higher score reflects higher depressive potential.

**Table 3:** Multivariate association of lipid parameters after adjustment for possible confounders with elevated SSI, CABS, HAM-D_17_ scales results (n = 203)*

	Suicidal ideation (SSI≥20)			Aggression (CABS≥22)			Depression (HAM-D_17_≥17)		
	OR	(95% CI)	*P*	OR	(95% CI)	*P*	OR	(95% CI)	*P*
Lipid parameters* (mmol/L):									
total cholesterol	0.09	(0.03-0.23)	<0.001	0.28	(0.14-0.59)	0.001	0.20	(0.08-0.48)	<0.001
LDL-C	1.27	(0.48-3.38)	0.629	1.05	(0.51-2.15)	0.894	1.47	(0.61-3.52)	0.391
HDL-C	0.97	(0.29-3.21)	0.956	1.73	(0.74-4.07)	0.209	1.15	(0.48-2.75)	0.751
triglycerides	0.90	(0.56-1.45)	0.669	0.90	(0.61-1.33)	0.604	0.99	(0.67-1.47)	0.970
**Confounders:**									
Age (years)	1.00	(0.94-1.06)	0.886	0.98	(0.93-1.03)	0.484	1.01	(0.96-1.07)	0.618
BMI	0.97	(0.82-1.16)	0.756	1.02	(0.88-1.19)	0.768	1.04	(0.88-1.22)	0.665
CAPS	0.98	(0.93-1.03)	0.356	0.98	(0.94-1.02)	0.357	0.98	(0.94-1.02)	0.363
Marital status									
married	1			1			1		
divorced	1.04	(0.35-3.12)	0.945	1.47	(0.62-3.51)	0.381	2.75	(1.12-6.78)	0.028
single	0.96	(0.17-5.49)	0.960	1.17	(0.32-4.32)	0.811	0.69	(0.20-2.35)	0.548
Educational level									
elementary school	1			1			1		
high school	0.91	(0.34-2.43)	0.852	0.81	(0.37-1.77)	0.598	1.21	(0.58-2.51)	0.619
>high school	4.45	(0.73-27.30)	0.104	0.58	(0.11-3.02)	0.515	3.80	(0.60-23.9)	0.156
Employment status									
full-time	1			1			1		
part-time	0.69	(0.06-8.11)	0.767	4.02	(0.71-22.91)	0.117	0.63	(0.12-3.29)	0.580
unemployed but able to work	1.48	(0.34-6.43)	0.605	0.63	(0.16-2.47)	0.506	0.83	(0.27-2.50)	0.733
sick-leave and unemployed unable to work	2.30	(0.45-11.69)	0.315	3.18	(0.78-12.91)	0.106	3.99	(0.96-16.7)	0.058
retired	1.13	(0.31-4.06)	0.853	3.22	(1.11-9.32)	0.031	0.99	(0.39-2.50)	0.977
Antidepressants									
SSRI	1.24	(0.36-4.24)	0.737	0.28	(0.11-0.75)	0.011	0.24	(0.08-0.76)	0.016
SNRI	1.95	(0.43-8.85)	0.386	0.59	(0.18-1.91)	0.379	0.32	(0.09-1.18)	0.087
NaSSA	1			1			1		

*Abbreviations: OR – odds ratio, multivariate binary logistic regression; 95% CI – 95% confidence interval; P – level of statistical significance; CAPS – Clinician-Administered PTSD Scale for DSM-IV, Current and Lifetime Diagnostic Version (38); a higher score reflects higher PTSD severity; LDL-C – low density lipoprotein cholesterol; HDL-C – high density lipoprotein cholesterol; SSRI – selective serotonin reuptake inhibitors; SNRI – serotonin norepinephrine reuptake inhibitors; NaSSA – noradrenergic and specific serotonergic antidepressants; SSI – Scale for Suicide Ideation (43); a higher score reflects higher suicidality potential; CABS – Corrigan Agitated Behavior Scale (41); a higher score reflects higher aggressive potential; HAM-D_17_ – Hamilton Depression Rating Scale (42); a higher score reflects higher depressive potential.

We found no significant moderating effect of PTSD severity on the association of total cholesterol and high SSI scale score. Association of total cholesterol and HAM-D_17_ score was significantly moderated by PTSD severity. At the 10th percentile of the CAPS scale result, association of total cholesterol and HAM-D_17_ score was not significant (odds ratio [OR] 0.46; 95% confidence interval [CI] 0.21-1.01; *P* = 0.053). At the 90th percentile of the CAPS scale result, association of total cholesterol and HAM-D_17_ score was significant (OR 0.15; 95% CI 0.07-0.34; *P* < 0.001). The Johnson-Neyman technique revealed that association of total cholesterol and HAM-D_17_ score was significant at CAPS value of 59.1, which corresponds to -1.5 CAPS standard deviations. Although the overall moderating effect of PTSD severity on the association of total cholesterol and CABS was not significant (*P* = 0.395), the Johnson-Neyman technique revealed that association of total cholesterol and the CABS score was significant if the CAPS score was lower than 83.9, which corresponds to 1.5 CAPS standard deviation.

## Discussion

The present study found an independent association between higher serum total cholesterol and lower odds for having higher suicidal ideation (SSI≥20), clinically significant aggression (CABS≥22), and at least moderate depressive symptoms (HAM-D_17_≥17) in male war veterans with PTSD without other psychiatric comorbidities. The association between total cholesterol and HAM-D_17_ scores and between total cholesterol and CABS scores was different in different PTSD severity groups. Consistently, previous studies reported higher prevalence of depressive symptoms in middle-aged men with lower cholesterol levels (≤4.5 mmol/L) than in men with cholesterol levels between 6 and 7 mmol/L (51). An American study failed to find a significant difference in the severity of depressive symptoms between the group of middle-aged adults with low (<3.9 mmol/L) and the group with total cholesterol ≥3.9 mmol/L (52). Various studies have found a negative association between total serum cholesterol and MDD, mostly associating lower levels of serum cholesterol with MDD (3-9,53,54). Higher serum cholesterol levels could be indirectly associated with lower levels of depression (depressive symptoms at a subsyndromal level) due to higher central serotonin activity than that expected in patients with lower serum cholesterol levels (25). Hypocholesterolemic status could be accompanied by more negative affective states at the clinical level (depressive symptoms more manifested, even in a full syndromal form – MDD) (20,25,28,51). However, in our PTSD population, neither low cholesterol levels nor MDD comorbidity were found.

In this study, a negative association between total cholesterol and CABS score was found. Literature data linked lower total cholesterol levels to higher rates of aggressive and violent behavior (11-13,55,56). In our study, higher serum total cholesterol levels were accompanied by less manifested agitation and aggressive behavioral patterns. Despite the lack of clinically significant manifestations of aggressive behaviors, patients with normal serum cholesterol levels in our sample manifested more aggressive behavioral patterns than those with borderline-high and high serum cholesterol levels.

Similarly to our results, other studies (23,24,26,27) also reported an association between low total cholesterol levels and suicidality, while one recent study found that only very low and very high cholesterol levels could be valid and reliable predictors of suicidal ideations incidence in elderly people (57).

In PTSD patients with high (>6.2 mmol/L) cholesterol levels and those with cholesterol levels within the upper range of borderline-high cholesterol category (cholesterol between 5.5-6.2 mmol/L), suicidal ideations were less pronounced than in patients with cholesterol below this value (5.5 mmol/L was the cut-off) but with each subsequent lowering of the serum cholesterol levels below the 5.5 mmol/L level, the SSI scores significantly increased. Our patients with normal serum total cholesterol levels had significantly more pronounced suicidal tendencies than those with borderline-high and high cholesterol levels. According to the trend described above, an even stronger association between low serum total cholesterol levels and high SSI scores could be expected in rare cases of low serum cholesterol. Serum cholesterol levels could represent cardiovascular risk factors, but it seems that they might have a protective effect against suicidal ideation in patients with PTSD. With a lowering of serum cholesterol values from high to normal levels, this protective effect weakens and suicidal tendencies increase, while with further lowering of serum cholesterol levels to hypocholesterolemic range, the increase becomes even more obvious.

Since some depressive symptoms may be attributed to PTSD clinical presentation, if the severity of PTSD symptoms reaches a critical level, their impact on the association between total cholesterol and depressive symptoms could became clinically significant. Previous studies focused on depression (MDD) comorbidity in combat-related PTSD suggest that PTSD may be a causal risk factor for subsequent depression (56). Depression comorbidity in PTSD has been associated with more severe and chronic symptomatology (58,59). Despite the fact that none of our patients had comorbid MDD, it seems that in patients with severe PTSD symptomatology (all of our patients were either markedly or moderately ill), PTSD itself could present a causal risk factor for subsequent depression (in our patients at subsyndromal level, depressive symptoms were present but not MDD comorbidity), partially mediated via serum cholesterol.

A limitation of our study was the cross-sectional design – we had a single fasting lipid measurement so the reliability of our independent variables may be insufficient. Also, without a control group, we might not have taken into account some confounding effects.

In conclusion, our results indicate a negative association between total cholesterol and higher suicidal ideation, clinically significant aggression, and at least moderate depressive symptoms. As a future research topic, we propose the analysis of a possible moderating effect of PTSD severity on the association of total cholesterol and the symptoms of depression. Monitoring of lipid level changes in PTSD patients could improve the therapeutic outcomes.
